# Association between blood lead and periodontitis among American adults: a cross-sectional study of the national health and nutrition examination survey

**DOI:** 10.3389/fphar.2024.1420613

**Published:** 2024-11-15

**Authors:** Yang Liu, Yuchuan Wu, Xiaolu Shi, Ye Tian, Shaobo Zhai, Zheng Yang, Shunli Chu

**Affiliations:** Department of Implantology, Hospital of Stomatology, Jilin University, Changchun, China

**Keywords:** periodontitis, lead, heavy metals, toxicity, national health and nutrition examination Survey, cross-sectional

## Abstract

**Background:**

Lead is persistent in the environment as a toxic substance and accumulates in the human body. Lead exposure has far-reaching harmful effects on all human systems and is widely recognized as a health and public health concern. Lead exposure poses a significant risk to oral health, as it destroys salivary glands and alveolar bone. It also induces oxidative stress which results in an immune response. Lead exposure appears to adversely affect periodontal tissues. Currently, the available evidence on the relationship between blood lead and periodontitis is insufficient and further research is necessary.

**Objective:**

In this study, the objectives were to clarify the association between blood lead and periodontitis, as well as to explore potential dose-response relationships between blood lead exposure and periodontitis, as well as to determine appropriate thresholds for the effects of blood lead on periodontitis.

**Methods:**

We conducted a cross-sectional study involving 8,550 participants with American adults aged 30 or older. blood Lead, periodontitis, age, sex, race, heart rate, education level, poverty index, marital status, body mass index, smoking status, alcohol drinking status, hypertension, diabetes and stroke. were collected from participants. Logistic regression, smooth curve fittingwere utilized to substantiate the research objectives.

**Results:**

There were a total of 8,550 participants of which 52.2% (4,467/8,550) had moderate/severe periodontitis. Compared with Q1 (0.15–0.93 ug/L), where blood lead levels were lower, the OR values for adjusted blood lead and periodontitis in Q2 (0.93–1.60 ug/L) and Q3 (1.60–61.29 ug/L) were 1.18 (95% CI: 1.12–1.25, *P* < 0.001) and 1.43 (95% CI: 1.34–1.52, *P* < 0.001). The association between blood lead levels and periodontitis exhibited a curve (non-linear, *P* < 0.001), with an inflection point of roughly 2.700 ug/L. The OR values for moderate/severe periodontitis in participants with blood lead levels <2.700ug/L was 1.318 (95% CI: 1.193–1.457, *P* < 0.001).

**Conclusion:**

Blood lead levels are positively associated with periodontitis. Blood lead levels increased by five units and were associated with an increase in moderate and severe periodontitis risk by 36%. There is a curvilinear relationship between blood lead and periodontitis with a threshold effect and an inflection point of approximately 2.7 ug/L.

## 1 Introduction

Periodontitis is a prevalent oral disease that is the leading cause of tooth loss in adults, involving up to 50% of the world’s population (Kinane et al., 2017; Chen et al., 2021). As a result of chronic periodontal inflammation caused by plaque, a host immune response occurs with the secretion of pro-inflammatory cytokines, resulting in the loss of periodontal attachment and reduced alveolar bone height, ultimately leading to the loss of teeth (Pan et al., 2019; Bai et al., 2021; Yang et al., 2023). At present, the primary treatment for periodontitis involves mechanical cleaning, root planning, surgery, as well as systemic or local administration of medications. These treatments, however, are associated with long treatment cycles, a high risk of recurrence, and drug resistance development (Luan et al., 2023). Considering the large number of people suffering from periodontitis and the poor prognosis of treatment, it is essential to emphasize the importance of preventative measures. The health risks of hazardous substances in the environment have always been an important public health issue, yet there are fewer studies on environmental factors as risk factors for periodontitis.

Heavy metals are common environmental pollutants that may pose a significant threat to human health through air, food and water (Liu et al., 2021; Spaur et al., 2022). Lead is a highly toxic heavy metal, and although it has been banned from many products, human exposure to lead remains a public health concern due to its persistence in the environment and its ability to accumulate in the body (Collin et al., 2022). Lead exposure has been associated with a wide range of adverse oral health effects. According to El-Said et al., workers exposed to lead for a long period of time experienced elevated blood lead levels, as well as significant negative effects on their oral health. These effects included an increase in periodontal disease prevalence, dental caries and dental abrasions (El-Said et al., 2008). Lead can damage the salivary glands and reduce their function (Hou et al., 2020). In addition, lead exposure induced an inflammatory response with alveolar bone resorption in rats (Terrizzi et al., 2013). By causing oxidative stress, lead exposure severely affects the immune system, making it more susceptible to bacteria (Metryka et al., 2018).

The search for a relationship between lead and periodontitis is of great importance in the field of health and public health. Currently, there is enough accumulated evidence to suggest that lead is strongly associated with periodontitis, however, the available evidence on the relationship between blood lead and periodontitis is insufficient. Therefore, to bridge this knowledge gap, we assessed the relationship between blood lead and periodontitis using data from the National Health and Nutrition Examination Survey (NHANES). More importantly the potential dose-response relationship between the two was explored.

## 2 Materials and methods

### 2.1 Data sources

This cross-sectional study utilized data from the National Health and Nutrition Examination Survey (NHANES), conducted by the Centers for Disease Control and Prevention between 2009 and 2014. NHANES is a comprehensive survey program designed to assess the health and nutritional status of noninstitutionalized individuals in the United States. The study collected demographic and health-related information through home visits, screenings, and laboratory tests conducted at mobile examination centers. Ethical approval was obtained from the National Center for Health Statistics Ethics Review Committee, and written informed consent was obtained from all participants. No additional Institutional Review Board approval was required for the secondary analyses. NHANES data is accessible through the NHANES website (http://www.cdc.gov/nchs/nhanes.htm) (accessed on 10 March 2024). For our study, we included individuals aged 30 years or older who completed interviews, while excluding those with missing data on periodontitis and blood lead levels. The current investigation adhered to the reporting guidelines stipulated by the Strengthening the Reporting of Observational Studies in Epidemiology framework.

### 2.2 Study design and population

**FIGURE 1:**
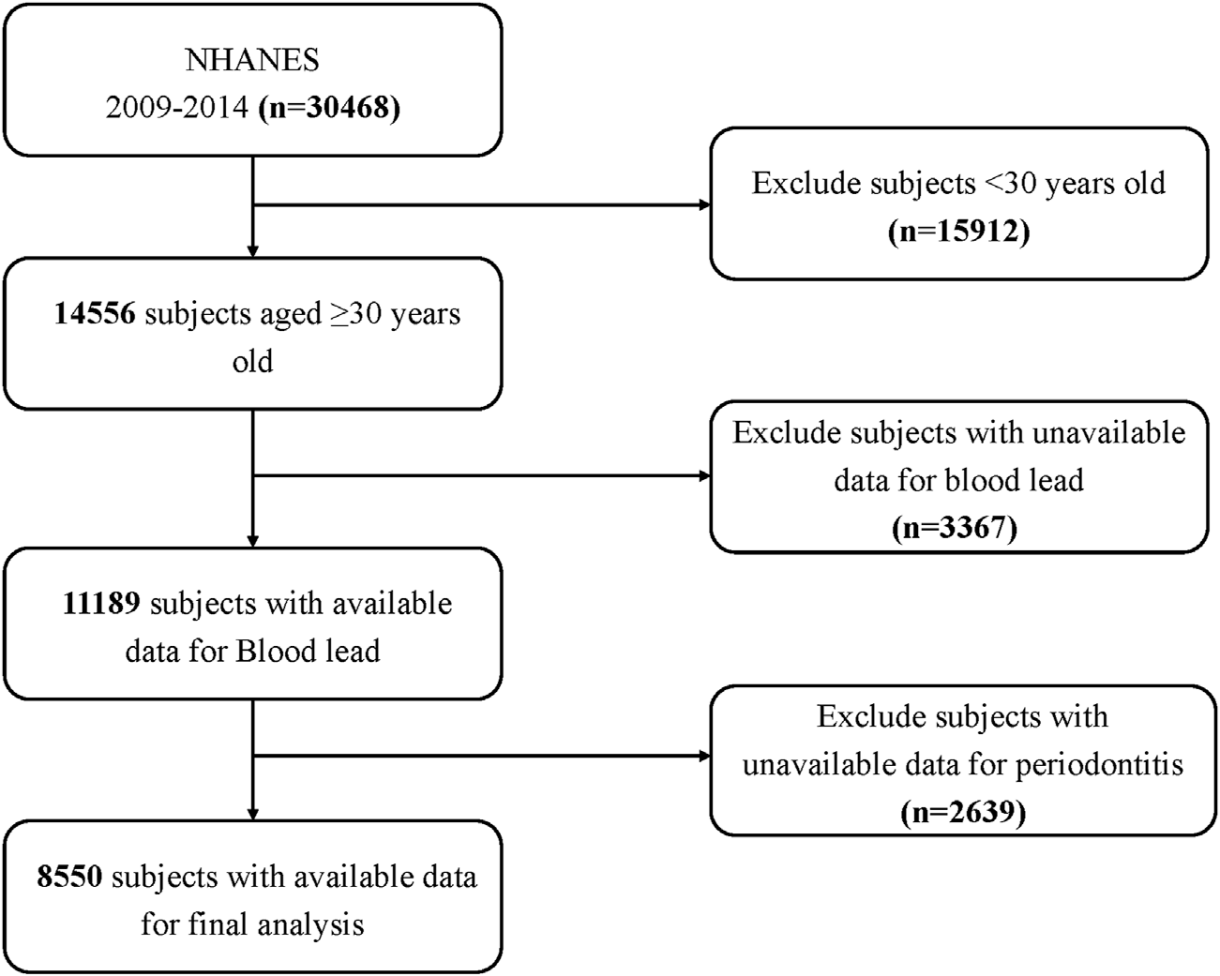
Schematic representation of the participant selection process and distribution of participant groups. NHANES, National Health and Nutrition Examination Survey.

Among the 30,468 participants who participated in the 2009–2014 NHANES, 15,912 subjects who were younger than 30 years were initially excluded, thereby leading to a population of 14,556 patients aged 30 or older. Then, we further excluded 3,367 individuals who did not have complete laboratory data on blood lead and 2,639 with no available examination data on periodontitis. Ultimately, a total of 8,550 subjects over 30 years old with complete data on blood lead and periodontitis were included in the study. Figure 1 shows the flowchart of the exclusion criteria.

### 2.3 Blood lead data

Blood heavy metal concentrations are considered to be a reliable indicator of actual exposure levels in the human body (Li et al., 2023). Blood lead levels measured in previous NHANES programs have played a crucial role in monitoring lead exposure in the United States. These data have been utilized to assess the prevalence of elevated blood lead levels, track the decline in lead exposure, advocate for the reduction of lead usage, and contribute to the development of national guidelines, standards, and interventions for lead poisoning prevention in the United States. The phlebotomists collected whole blood specimens at the NHANES Mobile Examination Center, where the samples were processed, frozen, and shipped to the CDC National Center for Environmental Health, where lead concentrations in whole blood samples are quantified by inductively coupled mass spectrometry. Instructions on specimen collection and processing are described in detail on the NHANES websites: https://www.cdc.gov/nchs/data/nhanes/nhanes_13_14/PbCd_H_MET.pdf.

### 2.4 Periodontitis data

The oral health examination was conducted by dental examiners, who were dentists licensed in at least one U.S. state. A health technician assisted in entering all examiner observations directly into a computerized data collection system. Two measurements were taken at 6 sites (mesio-, mid-, and distobuccal and mesio-, mid-, and distolingual) around each tooth other than the third molar. The measured attachment loss (AL) and probing depth (PD) data were evaluated according to the “CDC/AAP periodontitis case definitions for surveillance” to determine the severity of periodontitis (Eke et al., 2018; NHANES Survey Methods and Analytic Guidelines 2009, n. d.). The details are as follows:

Mild periodontitis: ≥2 interproximal sites with AL ≥3 mm and ≥2 interproximal sites with PD ≥ 4 mm (not on the same tooth) or one site with PD ≥ 5 mm;

Moderate periodontitis: ≥2 interproximal sites with AL ≥4 mm (not on the same tooth) or ≥2 interproximal sites with PD ≥ 5 mm (not on the same tooth); 

Severe periodontitis: ≥2 interproximal sites with AL ≥6 mm (not on the same tooth) and ≥1 interproximal site with PD ≥ 5 mm.

No periodontitis: no evidence of mild, moderate, or severe periodontitis.

The results were classified into two categories: no/mild periodontitis and moderate/severe periodontitis. Eligibility for the NHANES 2009–2014 periodontal examination was restricted to adults 30 years or older who had 1 or more natural teeth and no health conditions requiring antibiotic prophylaxis before periodontal probing.

### 2.5 Covariate

A directed acyclic graph (DAG) was plotted in DAGitty 3.1 to guide the modeling strategy and to determine the minimal sufficient adjustment set of variables ((MSAs, Supplementary Figure S1) of the variables. The MSAs consisted of 6 covariates: gender, age, race, education level, smoking status, and poverty index. The multivariate Logistic regression analysis was rerun based on the DAG results, and the covariates adjusted for model two were set to MSAs. In this study, a combination of questionnaires, physical examinations, and laboratory tests was employed to collect covariate data. The following covariates were included in the analysis: sex, age, race, educational level, marital status, poverty index, body mass index (BMI) (kg/m^2^), cotinine (ng/mL), alcohol drinking status, hypertension, diabetes, and stroke. Weight and height were measured by trained health technologists following the anthropometry procedure manual. The poverty index represents the ratio of household income to poverty. BMI was then calculated as weight in kilograms divided by height squared in meters. BMI data were classified into 3 categories including ≤25, 25.1–29.9, and ≥30 kg/m^2^. The quantification of serum cotinine content offers a more precise approach for assessing the extent of human exposure to tobacco smoke and evaluating the health effects associated with smoking (Lei et al., 2023). Therefore, serum cotinine levels were used to assess tobacco smoke exposure in this study. Alcohol status was determined based on subjects having at least 12 alcoholic drinks per year. The study investigated hypertension status and defined it using multiple criteria. Participants were first asked if they had ever been told they had high blood pressure, which represented the self-reported status of hypertension. Mean diastolic pressure greater than 90 mmHg and mean systolic pressure greater than 140 mmHg were then determined four times. Finally, participants with hypertension were identified based on their responses to the question “Because of your high blood pressure/hypertension, have you ever been told to take prescribed medicine?”. Diabetes was defined as self-report diabetes (Participants answering “yes” to the question, “Doctor told you have diabetes”) or current use of hypoglycemic agents (Participants answering “yes” to the question, “Take diabetic pills to lower blood sugar”) or a hemoglobin A1c level ≥6.5%. Participants with stroke were identified based on their responses to the question “Has a doctor or other health professional ever told you that you had a stroke?.”

### 2.6 Statistical analyses

This study involves a secondary analysis of publicly available datasets. Categorical variables were presented as proportions (%), while continuous variables were described using either the mean (standard deviation, SD) or median (interquartile range, IQR), depending on their distribution. To assess differences between groups, one-way analyses of variance (for variables with a normal distribution), Kruskal–Wallis tests (for variables with a skewed distribution), and chi-square tests (for categorical variables) were conducted. Logistic regression models were employed to estimate odds ratios (OR) and 95% confidence intervals (95% CIs) to examine the association between blood lead and periodontitis risk. Participants were categorized into three groups based on blood lead tertiles: Q1 (0.15–0.9 ug/L), Q2 (0.93–1.60 ug/L), and Q3 (1.60–61.29 ug/L), with the lowest tertile serving as the reference group. We performed a logistic regression analysis using blood lead as a continuous and categorical variable, respectively. Model 1 adjusts for age, sex, and race. Model 2 was further adjusted for education level and poverty index and marital status. Model 3 further adjusted for daily health status, including BMI, smoking status, and alcohol drinking status. Model 4: adjusted for history of illness, including hypertension, diabetes, and stroke, based on Model 3.

To further investigate the shape of the dose-response relationship between blood lead levels and periodontitis, a restricted cubic spline (RCS) regression analysis was carried out, utilizing four knots placed at the 5th, 35th, 65th, and 95th percentiles of blood lead. This analysis aimed to evaluate linearity and explore the dose-response relationship between blood lead and the risk of periodontitis after adjusting variables in Model 4. We used a Non-linearity tested by cubic spline term with smoothing to analyze the association threshold between blood lead and the risk of periodontitis after adjusting the variables in Model 4. The likelihood-ratio test and the bootstrap resampling method were used to determine inflection points. Moreover, logistic regression models were used to conduct interaction and subgroup analyses based on age, sex, race, education level, marital status, BMI, alcohol, hypertension, diabetes and stroke.

All analyses were performed using R Statistical Software (Version 4.2.2, http://www.R-project.org, The R Foundation) and Free Statistics analysis platform (Version 1.9, Beijing, China, http://www.clinicalscientists.cn/freestatistics). A two-tailed test was performed and *p* < 0.05 was considered statistically significant.

## 3 Results

### 3.1 Study population

A total of 30,468 participants completed interviews, of whom 15,912 were less than 30 years of age. We excluded those with missing blood lead data (n = 3,367), and those with missing periodontal screening data (n = 2,639). Ultimately, this cross-sectional study included 8,550 participants from NHANES Survey Methods and Analytic Guidelines, 2009 through 2014 in the analysis. Figure 1 depicts the comprehensive inclusion and exclusion procedure.

### 3.2 Baseline characteristics of participants

The baseline characteristics of included participants are presented in the Supplementary Material (Supplementary Table S1). Supplementary Table S1 depicts the baseline characteristics of all patients according to blood lead level tertiles. There were 4,467 patients (52.2%) with moderate/severe periodontitis. Previous studies have shown that the prevalence of periodontitis is as high as 50% globally (FDI Global Periodontal FDI Global Periodontal Health Project, 2017 NDA survey); the Global Burden of Disease (GBD) study shows that there are approximately 796 million cases of severe periodontitis (GBD, 2017 Disease and Injury Incidence and Prevalence Collaborators, 2018). The mean age of the study participants was (51.9 ± 14.2) years, of which 4,312 (50.4%) were female. Those with higher blood lead levels tended to be older; men; non-Hispanic white people/person or individual; better educated; moderate poverty index; married or living with a partner; moderate BMI; higher cotinine levels; alcohol consumption; and low prevalence of hypertension, diabetes, and stroke. In addition, a systematic review found significant gender differences in the prevalence of periodontitis, with males having a prevalence approximately 9% higher than females, and males appear to have a higher risk of destructive periodontal disease than females (Michelson et al., 2022). This was confirmed by the results of the present study, in which the prevalence of moderate/severe periodontitis was about 15.6% higher in men than in women.

### 3.3 Relationship between blood lead and periodontitis

**FIGURE 2:**
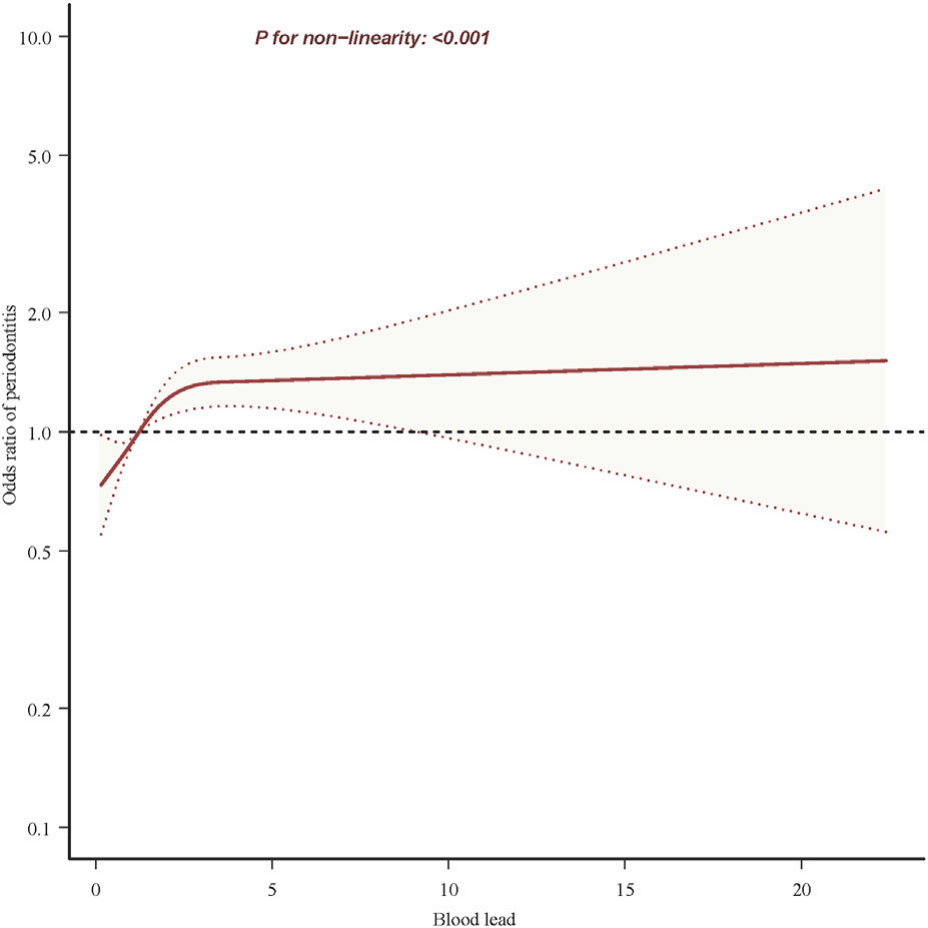
Association between blood lead and periodontitis risk. Solid and dashed lines represent the predicted value and 95% confidence intervals. They were adjusted for age, sex, race, heart rate, education level, poverty index, marital status, body mass index, smoking status, alcohol drinking status, hypertension, diabetes and stroke. Only 99.9% of the data is shown.

Univariate analyses showed that age, gender, race, education level, poverty index, marital status, smoking status, drinking status, hypertension, diabetes, and stroke were associated with periodontitis (Table 1). In the multivariate Logistic regression analysis (Table 2), when blood lead level was analyzed as a continuous variable, a significant independent positive association between blood lead level and risk of periodontitis was found in the non-adjusted crude model (OR: 4.63, 95% CI: 4.21–5.09; *p* < 0.001); When blood lead levels were analyzed in three equal parts, without adjusting for potential confounders, the ORs for blood lead levels and periodontitis were 1.98 (95% CI: 1.89–2.07, *p* < 0.001) for Q2 (0.93–1.60 ug/L) and 3.36 (95% CI:3.20–3.52, *p* < 0.001) for Q3 (1.60–61.29 ug/L) compared to Q1 (0.15–0.93 ug/L), where the blood lead level was lower, respectively. After adjusting for age, sex and race in model 1, the significance of the association between lead levels and periodontitis decreased (OR: 1.62, 95% CI: 1.50–1.76, *p* < 0.001). The significance of the association between lead level and periodontitis further decreased after further adjusting for education level, smoking status and poverty index in model 2 (OR: 1.11, 95% CI: 1.04–1.19; *p* = 0.002). After further adjusting for marital status, BMI and alcohol drinking status in model 3, the significance of the correlation between lead levels and periodontitis remained stable (OR: 1.13, 95% CI: 1.06–1.22; *p* < 0.001). Model 4 was further adjusted for hypertension, diabetes and stroke in the fully adjusted model 4, the risk of moderate/severe periodontitis increased by 15% when the blood lead concentration increased by 5 units (OR = 1.15, 95% CI: 1.07–1.23; *p* < 0.001). In the RCS, the relationship between blood lead levels and periodontitis was not linear but curved (nonlinear, *p* < 0.001) (Figure 2). In the threshold analysis, the OR for moderate/severe periodontitis was 1.318 (95% CI: 1.193–1.457, *p* < 0.001) for participants with blood lead levels <2.700 ug/L (Table 3). This means that for every 1 ug/L increase in blood lead level, the risk of developing moderate/severe periodontitis increased by 31.8%. There was no association between blood lead levels and periodontitis when blood lead levels were ≥2.700 ug/L (Table 3). This means that the risk of developing moderate/severe periodontitis no longer increases with increasing blood lead levels.

**TABLE 1:** Associations between blood lead and periodontitis risk.

Variable	OR (95%CI)	*p*-Value
blood lead, ug/L	1.36 (1.3–1.42)	<0.001
Age,years	1.05 (1.04–1.05)	<0.001
Sex, n (%)		
Male	1 (Reference)	
Female	0.5 (0.46–0.55)	<0.001
Race, n (%)		
Mexican American	1 (Reference)	
Other Hispanic	0.69 (0.58–0.82)	<0.001
Non-Hispanic White	0.51 (0.45–0.58)	<0.001
Non-Hispanic Black	1.02 (0.88–1.18)	0.800
Other Race	0.68 (0.57–0.8)	<0.001
Educational level, n (%)		
High school or less	1 (Reference)	
Some college	0.69 (0.6–0.79)	<0.001
College or above	0.33 (0.3–0.37)	<0.001
Poverty index, n (%)		
≤1.3	1 (Reference)	
1.4–3.5	0.70 (0.63–0.78)	<0.001
>3.5	0.34 (0.3–0.38)	<0.001
Marital status, n (%)		
Living alone	1 (Reference)	
Married or living with a partner	0.76 (0.69–0.83)	<0.001
BMI, n (%)		
≤25.0 kg/m^2^	1 (Reference)	
25.1–29.9 kg/m^2^	1.08 (0.97–1.2)	0.184
≥30.0 kg/m^2^	1.13 (1.01–1.25)	0.029
Cotinine, ng/mL	1 (1–1)	<0.001
Alcohol, n (%)		
No	1 (Reference)	
Yes	0.82 (0.74–0.9)	<0.001
Hypertension, n (%)		
No	1 (Reference)	
Yes	1.96 (1.75–2.19)	<0.001
Diabetes, n (%)		
No	1 (Reference)	
Yes	2.36 (2.08–2.67)	<0.001
Stroke, n (%)		
No	1 (Reference)	
Yes	2.08 (1.57–2.76)	<0.001

**TABLE 2:** Associations between blood lead and periodontitis risk in the multiple regression model.

Variable	In blood lead^a^		Blood lead levels quintiles (ug/L)		
	(n = 42,750)		Q1(n= 14,010)	Q2(n= 14,475)	Q3(n= 14,265)
	OR (95% CI)	*p*-value	OR (95% CI)	OR (95% CI)	OR (95% CI)
Unadjusted	4.63 (4.21–5.09)	<0.001	1.00 (ref)	1.98 (1.89–2.07)	3.36 (3.20–3.52)
Model 1^b^	1.62 (1.50–1.76)	<0.001	1.00 (ref)	1.30 (1.23–1.37)	1.77 (1.68–1.87)
Model 2^c^	1.11 (1.04–1.19)	0.002	1.00 (ref)	1.13 (1.07–1.20)	1.34 (1.26–1.42)
Model 3^d^	1.13 (1.06–1.22)	<0.001	1.00 (ref)	1.16 (1.10–1.23)	1.39 (1.31–1.47)
Model 4^e^	1.15 (1.07–1.23)	<0.001	1.00 (ref)	1.18 (1.12–1.25)	1.43 (1.34–1.52)

^a^ Blood Lead was entered as a continuous variable per 5 ug/L increase.

^b^ Model 1: adjusted for age, sex and race.

^c^ Model 2: adjusted as for model 1, additionally adjusted for education level, smoking status and poverty index.

^d^ Model 3: adjusted as for model 2, additionally adjusted for marital status, body mass index (BMI) and alcohol drinking status.

^e^ Model 4: adjusted as for model 3, additionally adjusted for hypertension, diabetes and stroke.

OR, odds ratio; 95% CI, 95% confidence interval.

**TABLE 3:** Threshold effect analysis of the relationship of blood lead with periodontitis risk.

Item	OR (95%CI)	*p*-Value
Blood lead <2.700 ug/L	1.318 (1.193–1.457)	<0.001
Blood lead ≥2.700 ug/L	1.010 (0.950–1.074)	0.751
Likelihood Ratio test		<0.001

Adjusted for age, sex, race, heart rate, education level, poverty index, marital status, body mass index, smoking status, alcohol drinking status, hypertension, diabetes and stroke. OR, odds ratio; 95% CI, 95% confidence interval.

### 3.4 Stratified analyses based on additional variables

Stratified analyses were conducted in several subgroups to assess the potential modifications in the relationship between blood lead levels and periodontitis. No significant interactions were found in any of the subgroups after stratification according to age, sex, race, education level, poverty index, marital status, and body mass index, as well as whether or not alcohol was consumed, or whether or not one had hypertension or diabetes, or had had a stroke (Supplementary Figure S2).

### 3.5 Sensitivity analysis

After excluding 280 individuals with extreme blood lead levels (Blood lead >10.000 ug/L) for sensitivity analysis, the relationship between blood lead levels and periodontitis remained stable. After adjusting for all covariates, the corrected ORs for blood lead levels and periodontitis were 1.18 (95% CI: 1.12–1.25, *p* < 0.001) and 1.42 (95% CI: 1.34–1.51, *p* < 0.001) for Q2 and Q3, respectively, compared to Q1, the individuals with lower blood lead (Supplementary Table S2).

## 4 Discussion

This large cross-sectional study of U.S. adults aged 30 or older showed an independent association between blood lead levels and periodontitis after correcting for potential confounders. In addition, there was a curvilinear relationship between blood lead levels and periodontitis with an inflection point of 2.7 ug/L and a threshold effect. Some differences in the association between blood lead and periodontitis were found in the stratified analysis; however, these differences were not statistically significant.

Early on, it was shown that bone loss in advanced periodontitis was positively correlated with blood lead levels (Dye et al., 2002). Currently, studies have reported the association between blood lead and periodontitis in the Korean population (Y and Bk, 2013). In addition, a study published in 2007 using the NHANES III database also showed a positive correlation between blood lead levels and periodontitis (Mc et al., 2007). However, in 2012 the definition of periodontitis has changed since then and has been categorized as mild, severe, and severe according to its severity, so it is necessary to re-examine the study based on the new diagnostic criteria (Eke et al., 2012). In this study, we chose to use NHANES 2009–2014 data to study a population of American adults aged 30 or older, and the results confirmed that the association between blood lead and periodontitis was significant and stable. More importantly, we determined a curvilinear relationship between blood lead and periodontitis using appropriate statistical methods rather than a simple straight line relationship. In addition, we found inflection points for smooth curve fitting rather than artificial grouping. These results could prompt physicians to focus on the heavy metal lead as a risk factor for periodontitis and prompt patients to prevent lead exposure.

To our knowledge, this is the first time that a threshold effect between blood lead and periodontitis has been identified. The adverse effects of increased blood lead levels on periodontitis appear to peak in populations with higher blood lead levels. Specifically, in patients with blood lead levels <2.7 ug/L, the risk of having moderate-to-severe periodontitis increased with increasing blood lead levels, whereas in patients with blood lead levels ≥2.7 ug/L, the risk of moderate-to-severe periodontitis no longer increased with increasing blood lead levels. Lead can enter the atmosphere, soil and water bodies through a variety of sources, including industrial activities (Gao et al., 2023), mining and smelting operations (Mathee et al., 2022) and the use of products containing lead (Chen et al., 2022). Lead may be absorbed by plants and ultimately affect human health through the consumption of food and drinking water. In addition, the use of lead paint (Cissé, 2023), corrosion of lead plumbing, and exposure to lead-containing consumer products are all sources of blood lead (Childhood Lead Poisoning Prevention: Sources of Lead Exposure, 2023). Although there are ongoing public policies to reduce human lead exposure and they have had demonstrable results, human exposure to lead remains a public health problem due to its persistence in the environment, its ability to accumulate in the body, and the severity of the harm caused by lead toxicity (Roy et al., 2023).

There are some limitations to consider. First, this study classifies periodontitis into mild, moderate, and severe categories based on probing depth and clinical attachment loss (Eke et al., 2012). Currently, the American Academy of Periodontology has established a new classification system for periodontitis as of 2017 (Morales et al., 2022). However, due to the limitations of the NHANES database, we are unable to incorporate this new classification into our research. Second, blood lead and periodontitis data were only collected in NHANES between 2009 and 2014. This precluded further validation using NHANES data from a different time period. Third, even with regression modeling and stratified analyses, residual confounding effects of unmeasured or unknown factors could not be completely excluded. Fourth, the current findings are from a survey of U.S. adults aged 30 years or older in NHANES, and further investigation is needed to determine whether they can be generalized to other populations. Finally, due to the inherent limitations of cross-sectional studies, a causal relationship between blood lead and periodontitis could not be established. Future studies may consider the use of more rigorous study designs, broader sample selection, more comprehensive confounding considerations, more in-depth mechanistic studies, and more advanced statistical methods to more accurately assess the relationship between blood lead and periodontitis. In addition to the association between blood lead and periodontitis, we can further explore other risk factors that may influence periodontitis in the future.

## 5 Conclusion

In conclusion, blood lead was positively associated with periodontitis risk in this large, cross-sectional, population-based study that included 8,550 Americans aged 30–79 years in the 2009–2014 NHANES survey; the risk of moderate/severe periodontitis increased by 36% when blood lead concentrations increased by 5 units. There is a curvilinear relationship between blood lead and periodontitis with a threshold effect and an inflection point of approximately 2.7 ug/L. The results of this study reveal risk factors associated with periodontitis, and the curvilinear relationship may shed light on future targets for interventions aimed at reducing its incidence.

## Data Availability

Publicly available datasets were analyzed in this study. This data can be found here: https://www.cdc.gov/nchs/nhanes/index.htm. Specifically containing data from three cycles of the nhanes database 2009–2010, 2011–2012, and 2013–2014, the data can be found in the following files: demographic variables and sample weights, body measures, blood pressure, oral health-periodontal, cotinine and hydroxycotinine-serum, lead, cadmium, total mercury, selenium, and manganese-blood, alcohol use, blood pressure & cholesterol, diabetes, medical conditions.
