# Supplementary material for: Association between blood lead and periodontitis among American adults: a cross-sectional study of the national health and nutrition examination survey
Source: Front Pharmacol. 2024 Nov 15;15:1420613. doi: 10.3389/fphar.2024.1420613 (PMC11604452; doi:10.3389/fphar.2024.1420613)
Supplement: Supplementary file 1 [file DataSheet1.docx]

Supplementary Material

| **Variables** | **Total(n = 8550)** | **Blood Lead quartiles (pg/mL)** | | | ***P* value** |
| --- | --- | --- | --- | --- | --- |
|  |  | **Q1 (n=2802)** | **Q2 (n=2895)** | **Q3 (n=2853)** |  |
| Age,years | 51.9 ± 14.2 | 45.3 ± 12.6 | 53.2 ± 13.7 | 57.1 ± 13.7 | < 0.001 |
| Sex, n (%) |  |  |  |  | < 0.001 |
| Male | 4238 (49.6) | 954 (34) | 1475 (50.9) | 1809 (63.4) |  |
| Female | 4312 (50.4) | 1848 (66.0) | 1420 (49.1) | 1044 (36.6) |  |
| Race, n (%) |  |  |  |  | < 0.001 |
| Mexican American | 1242 (14.5) | 404 (14.4) | 416 (14.4) | 422 (14.8) |  |
| Other Hispanic | 866 (10.1) | 353 (12.6) | 307 (10.6) | 206 (7.2) |  |
| Non-Hispanic White | 3705 (43.3) | 1273 (45.4) | 1232 (42.6) | 1200 (42.1) |  |
| Non-Hispanic Black | 1745 (20.4) | 501 (17.9) | 579 (20.0) | 665 (23.3) |  |
| Other Race | 992 (11.6) | 271 (9.7) | 361 (12.5) | 360 (12.6) |  |
| Educational level, n (%) |  |  |  |  | < 0.001 |
| High school or less | 2039 (23.8) | 520 (18.6) | 675 (23.3) | 844 (29.6) |  |
| Some college | 1855 (21.7) | 565 (20.2) | 633 (21.9) | 657 (23.0) |  |
| College or above | 4656 (54.5) | 1717 (61.3) | 1587 (54.8) | 1352 (47.4) |  |
| Poverty index, n (%) |  |  |  |  | < 0.001 |
| ≤1.3 | 2575 (30.1) | 795 (28.4) | 824 (28.5) | 956 (33.5) |  |
| 1.4-3.5 | 3066 (35.9) | 1008 (36.0) | 1052 (36.3) | 1006 (35.3) |  |
| >3.5 | 2909 (34.0) | 999 (35.7) | 1019 (35.2) | 891 (31.2) |  |
| Marital status, n (%) |  |  |  |  | 0.049 |
| Living alone | 2997 (35.1) | 935 (33.4) | 1022 (35.3) | 1040 (36.5) |  |
| Married or living  with a partner | 5553 (64.9) | 1867 (66.6) | 1873 (64.7) | 1813 (63.5) |  |
| BMI, n (%) |  |  |  |  | < 0.001 |
| ≤25.0 kg/m2 | 2324 (27.2) | 660 (23.6) | 766 (26.5) | 898 (31.5) |  |
| 25.1-29.9kg/m2 | 2934 (34.3) | 831 (29.7) | 1045 (36.1) | 1058 (37.1) |  |
| ≥30.0kg/m2 | 3292 (38.5) | 1311 (46.8) | 1084 (37.4) | 897 (31.4) |  |
| Cotinine, ng/mL | 0.0 (0.0, 1.4) | 0.0 (0.0, 0.1) | 0.0 (0.0, 0.6) | 0.1 (0.0, 112.0) | < 0.001 |
| Alcohol, n (%) |  |  |  |  | < 0.001 |
| No | 2311 (27.0) | 882 (31.5) | 797 (27.5) | 632 (22.2) |  |
| Yes | 6239 (73.0) | 1920 (68.5) | 2098 (72.5) | 2221 (77.8) |  |
| Hypertension, n (%) |  |  |  |  | < 0.001 |
| No | 6924 (81.0) | 2412 (86.1) | 2348 (81.1) | 2164 (75.8) |  |
| Yes | 1626 (19.0) | 390 (13.9) | 547 (18.9) | 689 (24.2) |  |
| Diabetes, n (%) |  |  |  |  | 0.550 |
| No | 7174 (83.9) | 2349 (83.8) | 2415 (83.4) | 2410 (84.5) |  |
| Yes | 1376 (16.1) | 453 (16.2) | 480 (16.6) | 443 (15.5) |  |
| Stroke, n (%) |  |  |  |  | < 0.001 |
| No | 8317 (97.3) | 2760 (98.5) | 2818 (97.3) | 2739 (96) |  |
| Yes | 233 ( 2.7) | 42 (1.5) | 77 (2.7) | 114 (4.0) |  |
| Periodontitis, n (%) |  |  |  |  | < 0.001 |
| Non/mild  periodontitis | 4083 (47.8) | 1771 (63.2) | 1346 (46.5) | 966 (33.9) |  |
| Moderate/severe periodontitis | 4467 (52.2) | 1031 (36.8) | 1549 (53.5) | 1887 (66.1) |  |

**Supplementary Table 1.** Baseline characteristics of the study participants

| **Variable** | **In Blood Lead^a^** | |  | **Blood Lead levels quintiles (ug/L)** | | |
| --- | --- | --- | --- | --- | --- | --- |
|  | **(n = 42470)** | |  | **Q1(n=14010)** | **Q2(n=14075)** | **Q3(n=14385)** |
|  | **OR (95% CI)** | ***P* value** |  | **OR (95% CI)** | **OR (95% CI)** | **OR (95% CI)** |
| **Unadjusted** | 12.4 (11.07~13.88) | <0.001 |  | 1.00 (ref) | 1.97 (1.88~2.07) | 3.30 (3.15~3.47) |
| **Model 1^b^** | 3.41 (3.04~3.81) | <0.001 |  | 1.00 (ref) | 1.30 (1.23~1.37) | 1.76 (1.66~1.86) |
| **Model 2^c^** | 1.88 (1.67~2.11) | 0.002 |  | 1.00 (ref) | 1.14 (1.07~1.20) | 1.34 (1.26~1.42) |
| **Model 3^d^** | 2.00 (1.77~2.25) | <0.001 |  | 1.00 (ref) | 1.16 (1.10~1.23) | 1.39 (1.31~1.47) |
| **Model 4^e^** | 2.08 (1.84~2.34) | <0.001 |  | 1.00 (ref) | 1.18 (1.12~1.25) | 1.42 (1.34~1.51) |

**Supplementary Table 2.** Association between blood lead and periodontitis risk in participants with extreme energy intake was not included.

^a^ Blood Lead was entered as a continuous variable per 5 ug/L increase

^b^ Model 1: adjusted for age, sex and race

^c^ Model 2: adjusted as for model 1, additionally adjusted for education level, smoking status and poverty index

^d^ Model 3: adjusted as for model 2, additionally adjusted for marital status, body mass index (BMI) and alcohol drinking status

^e^ Model 4: adjusted as for model 3, additionally adjusted for hypertension, diabetes and stroke

OR, odds ratio; 95% CI, 95% confidence interval.


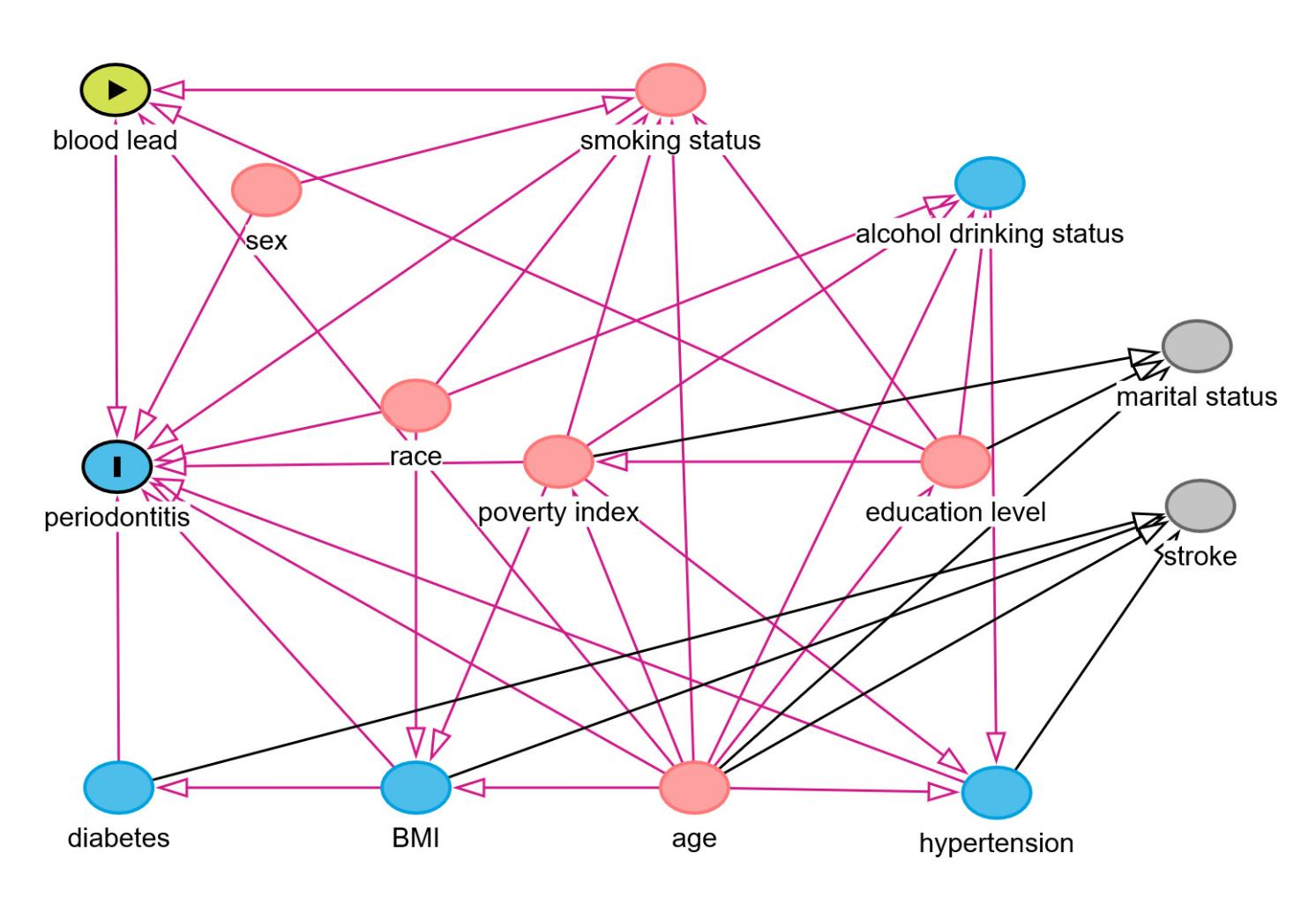


Supplementary Figure 1. The association between blood lead and periodontitis based on a directed acyclic graph.


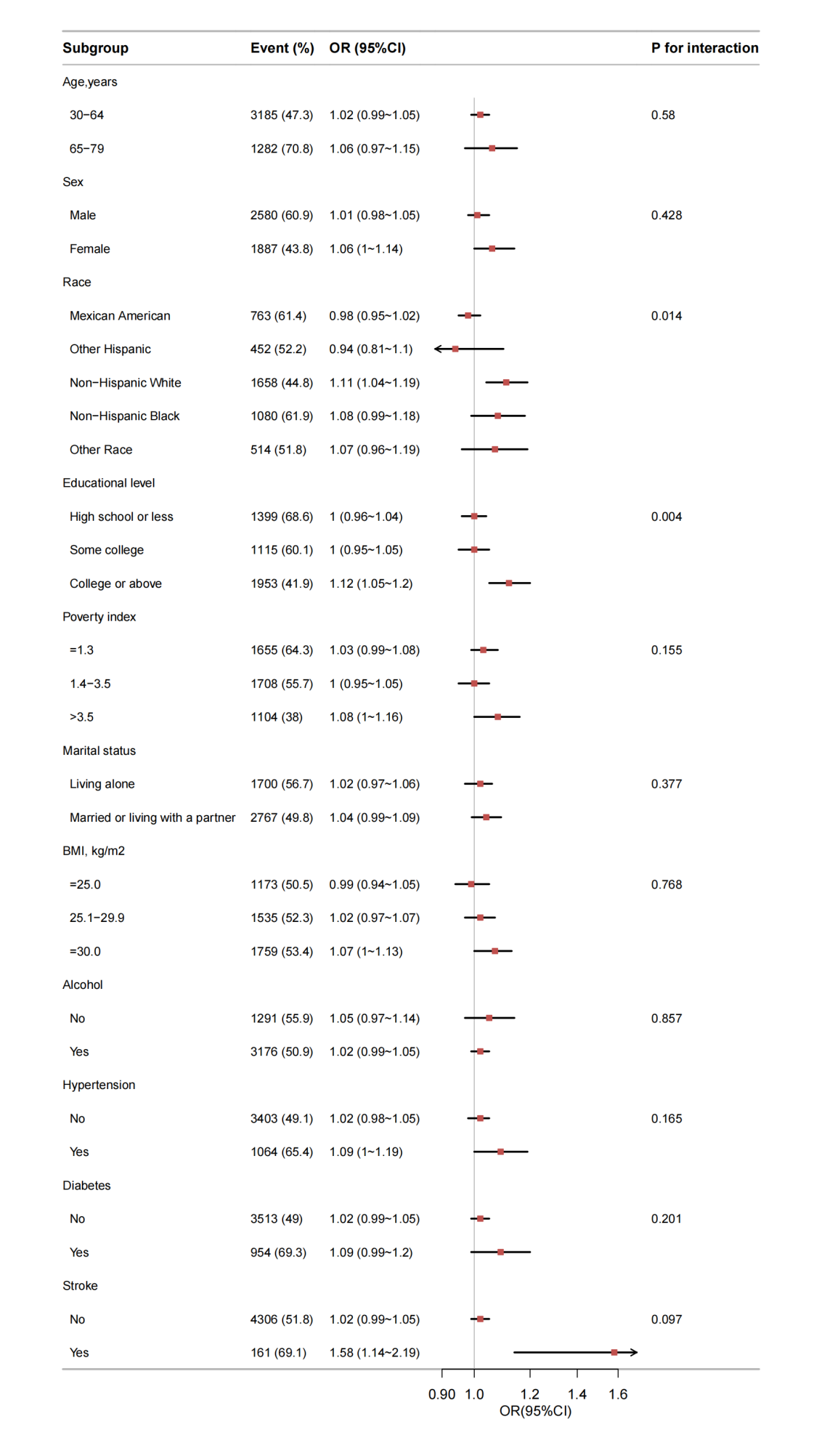


**Supplementary Figure 2.** Associations between blood lead with periodontitis risk in different subgroups. Except for the stratification component itself, each stratification factor was adjusted for age, sex, race, heart rate, education level, poverty index, marital status, body mass index, smoking status, alcohol drinking status, hypertension, diabetes and stroke. OR, odds ratio; 95% CI, 95% confidence interval.
